# Artikulation von Suizidwünschen von Patient:innen im Behandlungskontext – Erfahrungen und Unterstützungsbedarf von Krankenhausmitarbeitenden

**DOI:** 10.1007/s00103-026-04195-w

**Published:** 2026-02-09

**Authors:** Anna-Christin Willert, Julia Rohe, Anja Prass, Johann Ahn, Susanne Michl

**Affiliations:** 1 Klinik für Neurologie mit Experimenteller Neurologie, Campus Virchow-Klinikum, Charité – Universitätsmedizin Berlin, Augustenburger Platz 1, 13353 Berlin, Deutschland; 2 Geschäftsstelle Klinisches Ethikkomitee, Charité – Universitätsmedizin Berlin, Berlin, Deutschland; 3 Klinik für Pädiatrie m.S. Pneumologie, Immunologie und Intensivmedizin, Charité – Universitätsmedizin Berlin, Berlin, Deutschland; 4 Medizinische Klinik m.S. Hämatologie, Onkologie und Tumorimmunologie, Campus Virchow-Klinikum, Charité – Universitätsmedizin Berlin, Berlin, Deutschland; 5 Institut für Geschichte und Ethik in der Medizin, Charité – Universitätsmedizin Berlin, Berlin, Deutschland

**Keywords:** Assistierter Suizid, Suizidprävention, Fort- und Weiterbildung, Suizidpräventionsstrategie, Suicidal ideation, Suicide prevention, Continuing education, Suicide prevention strategy

## Abstract

**Hintergrund:**

Das Thema „assistierter Suizid“ ist seit dem Urteil des Bundesverfassungsgerichts vom Februar 2020 in Politik, Gesellschaft und Medizin sehr relevant. Dies könnte dazu führen, dass Patient:innen häufiger Suizidwünsche äußern. Ziele der Studie sind 1) die Ermittlung der Erfahrungen von Krankenhausmitarbeitenden mit Suizidwünschen im Behandlungskontext sowie ihres Kenntnisstands zu Suizidpräventionsangeboten und 2) die Evaluation des Umgangs mit Suizidwünschen sowie der Unterstützungsbedarfe.

**Methoden:**

Onlinebefragung aller Mitarbeitenden eines Universitätskrankenhauses der Maximalversorgung von Februar bis März 2024. Die Auswertung erfolgte quantitativ, Freitextangaben wurden inhaltsanalytisch untersucht.

**Ergebnisse:**

741 Mitarbeitende unterschiedlicher Berufsgruppen nahmen teil. Es gaben 83 % an, im Behandlungskontext 1‑ bis >5-mal einen konkreten Suizidwunsch gehört zu haben; 42 % wurden bereits um konkrete Suizidassistenz gebeten. Nur 33 % kannten Angebote zur Suizidprävention. Als stärkende Ressourcen im Umgang mit Suizidwünschen wurden „Menschenkenntnis“ (78 %) und das „Hinzuziehen anderer Berufsgruppen“ (74 %) genannt. Gewünscht wurden als institutionelle Unterstützung „Fortbildungen“ (68 %), „Kommunikationsleitfäden“ (55 %) und „Supervision“ (36 %).

**Diskussion:**

Die Mehrheit der teilnehmenden Krankenhausmitarbeitenden hat Erfahrungen mit Suizidwünschen und ein relevanter Anteil wurde bereits um Suizidassistenz gebeten. Präventionsangebote sind jedoch weniger bekannt. Angesichts erwarteter Zunahmen von Suizidwunschäußerungen sind Strategien zur Bereitstellung von Suizidpräventions- und Unterstützungsangeboten essenziell.

**Zusatzmaterial online:**

Zusätzliche Informationen sind in der Online-Version dieses Artikels (10.1007/s00103-026-04195-w) enthalten.

## Abstract (en)

**Background:**

The ruling of the German Federal Constitutional Court of February 2020 might reinforce patients to be more straightforward about suicidal ideation. This study aims 1) to evaluate experiences of healthcare professionals with suicidal ideation within the therapeutic context and their awareness of suicide prevention services and 2) to assess their resources and need for institutional support.

**Methods:**

Online survey of all healthcare professionals of an academic tertiary hospital between February and March 2024. The analysis was conducted quantitatively; free-text responses were analysed for content.

**Results:**

Of the 741 healthcare professionals who participated, 83% experienced a situation of suicidal ideation within the therapeutic context 1 to > 5 times; 42% were asked for suicide assistance. Only 33% knew specific suicide prevention services. As resources to cope with suicidal ideation, they named “knowledge of human nature” (78%) and “interprofessional consultation” (74%). The need for “training” (68%), “communication guidelines” (55%) and “supervision” (36%) was outlined.

**Discussion:**

Most participating healthcare professionals experienced suicidal ideation within the therapeutic context, and a relevant portion was asked for suicide assistance. However, suicide prevention services were less well known. As suicide wishes may increase in hospitals, strategies to provide prevention information and hospital staff support are essential.

## Hintergrund

Seit dem Urteil des Bundesverfassungsgerichts vom 26.02.2020, welches das Verbot der geschäftsmäßigen Förderung der Selbsttötung (§ 217 StGB) für nichtig erklärte, wird eine Neuregelung der Suizidhilfe in Politik, Medien, medizinischen Fachgesellschaften sowie Berufsvereinigungen kontrovers diskutiert [1]. Im Urteil formulieren die Richter:innen ein Recht auf selbstbestimmtes Sterben als Ausdruck persönlicher Autonomie und Teil des allgemeinen Persönlichkeitsrechts. Es ist zu erwarten, dass Suizidwünsche nun häufiger geäußert werden bzw. direkter und nachdrücklicher in stationären wie ambulanten Behandlungskontexten nach Suizidassistenz gefragt wird [2].

Unter „Todeswünschen“ wird das Spektrum zwischen Hoffnung auf ein rasches Versterben bis zu konkreten Suizidplänen verstanden [2]. Ein „Suizidwunsch“ liegt vor, wenn der Wunsch besteht, das eigene Leben selbst zu beenden. Ein „assistierter Suizid“ meint eine „Selbsttötung mit Beihilfe“. Die sterbewillige Person nimmt selbstständig beispielsweise eine Substanz zur Selbsttötung zu sich. Eine andere Person leistet hierzu einen Beitrag.

Diese Studie zielt darauf ab, die Erfahrungen von Mitarbeitenden einer maximalversorgenden Universitätsklinik mit Suizidwünschen von Patient:innen im Behandlungskontext zu untersuchen und die Unterstützungsbedarfe zu ermitteln. In der bestehenden Literatur wird der Bedarf an Unterstützung für Krankenhausmitarbeitende bislang nicht ausreichend berücksichtigt.

## Methoden

Die Erfahrungen und Unterstützungsbedarfe der Mitarbeitenden wurden mittels eines offenen elektronischen Fragebogens vom 19.02.– 20.03.2024 an der Charité – Universitätsmedizin Berlin erhoben. Die Darlegung der Methoden erfolgt anhand der „Checklist for Reporting Results of Internet E‑Surveys“ (CHERRIES; [3]).

Die Charité hat ca. 140.000 stationäre und 800.000 ambulante Behandlungsfälle im Jahr. Im Beobachtungszeitraum sind insgesamt 10.969 Dienst-aktive Mitarbeiter:innen an der Charité beschäftigt. Hiervon gehören 3091 dem ärztlichen Dienst an, 7443 dem Pflegedienst, 233 sind Psycholog:innen, 55 Mitarbeitende arbeiten im Sozialdienst und 157 in der Physio‑/Ergotherapie. Zusätzlich sind 11 Seelsorger:innen aktiv. Alle Berufsgruppen (Pflege, Ärzteschaft, Psychologie, Physiotherapie, Ergotherapie, soziale Arbeit, Seelsorge, andere) wurden per E‑Mail und über eine Intranet-Meldung zur freiwilligen, anonymen Teilnahme eingeladen; der Teilnahmelink war entsprechend im Anschreiben der E‑Mail sowie im Intranet für Mitarbeitende verfügbar. Zur Durchführung wurde die Umfragesoftware EvaSys (V10.0 (2621); evasys GmbH, Lüneburg, NI, Deutschland) genutzt. Vor Einholen eines informierten Einverständnisses erhielten die Teilnehmenden zu Beginn des Fragebogens Informationen zur Studie sowie zum Datenschutz. Zur Wahrung der Anonymität wurden keine personenbezogenen Daten (Alter, Geschlecht, Tätigkeitsort) erfasst. Die Teilnehmenden wurden zudem gebeten keine personenbezogenen Daten in Freitextfeldern anzugeben. Die anschließende Veröffentlichung der Ergebnisse sowie die Schaffung spezifischer Unterstützungsangebote für die Mitarbeitenden wurden in Aussicht gestellt.

Die Onlinebefragung umfasste 10 Items zu den Themen „Erfahrung mit Suizidwünschen im Behandlungskontext“, „Ressourcen im Umgang mit Suizidwünschen“ und „Bedarf an institutioneller Unterstützung zum Thema Suizidwünsche im Allgemeinen sowie spezifisch zum Urteil des Bundesverfassungsgerichts, den zugehörigen Gesetzesentwürfen und der gesellschaftlichen Debatte“, „persönliche Positionierung in der Diskussion zum assistierten Suizid“ sowie „Kenntnisse von Angeboten der Suizidprävention“. Der Fragebogen ist im Onlinematerial zu finden. Die Fragen wurden durch eine interdisziplinär besetzte Arbeitsgruppe des Klinischen Ethikkomitees der Charité erarbeitet. Entsprechend den Umfragezielen wurden durch Literaturrecherche, Expertenwissen und Gruppendiskussionen zentrale Themen definiert und zu diesen Fragen formuliert. Prätests mit Kommentarmöglichkeit erfolgten zur Prüfung von Verständlichkeit, Durchführbarkeit und Inhalt sowohl innerhalb der Arbeitsgruppe des Klinischen Ethikkomitees als auch durch 6 unabhängige Personen aus dem pflegerischen und ärztlichen Dienst. Die erhaltenen Rückmeldungen wurden in der Arbeitsgruppe diskutiert und entsprechend eingearbeitet. Abschließend erfolgte die Abstimmung mit dem Datenschutz und dem Personalrat.

Eine Randomisierung, Alternierung oder Gewichtung der Items erfolgte nicht. Zwei Filterfragen wurden angewendet, um die Fragen an die Erfahrungen und Kenntnisse der Mitarbeitenden anzupassen. Pflichtfelder bestanden nicht; jedes Item hatte eine „Keine-Angabe“-Option. Den Teilnehmenden war es möglich, ihre Antworten vor dem Absenden zu überprüfen. Insgesamt umfasste die Onlinebefragung 3 Seiten. Auf Cookies, einen IP-Check oder eine Log-file-Analyse wurde bei PCs mit Mehrfachnutzung am Arbeitsplatz verzichtet. Bei verschiedenen Dienstmodellen wurde die Uhrzeit der Teilnahme nicht berücksichtigt.

Die Auswertung erfolgte mit SPSS Statistics (IBM Inc., Armonk, NY, USA) und Microsoft Excel (Version 16.94; Microsoft Corporation, Redmond, WA, USA). Quantitative Daten sind in Prozent (Anzahl *n*/Gesamtanzahl der Antworten des Items) angegeben. Fehlende Werte wurden bei der jeweiligen Analyse ausgeschlossen. Die Freitexte wurden einer induktiven, zusammenfassenden qualitativen Inhaltsanalyse unterzogen [4].

## Ergebnisse

**Abb. 1:**
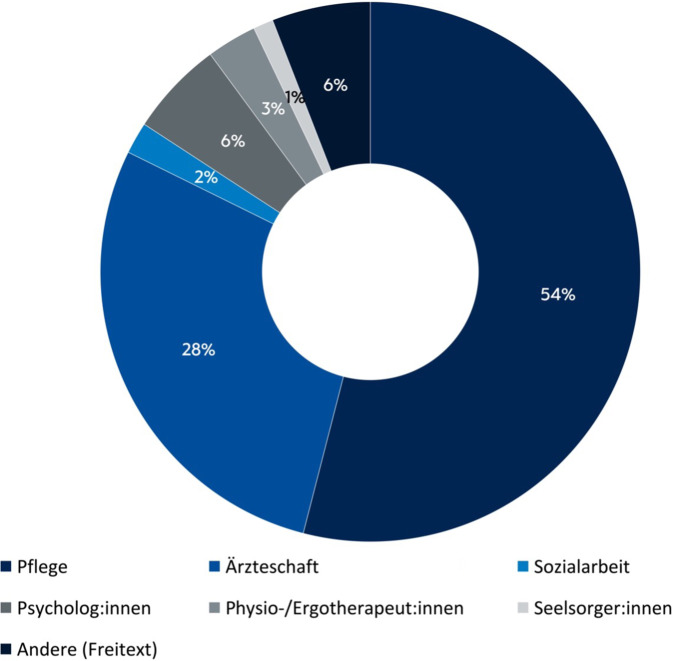
Teilnehmende Berufsgruppen

Insgesamt nahmen 741 von insgesamt 10.980 Dienst-aktiven Mitarbeitenden an der Befragung teil (Abb. 1).

### Suizidwünsche im Behandlungskontext.

Gegenüber 83 % (616/741) der Mitarbeitenden wurde während ihrer beruflichen Tätigkeit 1‑ bis >5-mal ein Suizidwunsch geäußert. 15 % (109/741) sind noch nicht mit einem Suizidwunsch in Berührung gekommen, 2 % machten keine Angaben (16/741).

Um konkrete Unterstützung bei der Durchführung eines Suizids wurden bereits 42 % (310/736) der Mitarbeitenden 1‑ bis >5-mal gebeten; 55 % (407/736) wurden noch nicht mit einem Gesuch nach Suizidassistenz konfrontiert. Keine Angaben machten 3 % (19/736).

### Ressourcen der Mitarbeitenden.

Mitarbeitende, die bereits Erfahrungen mit Suizidwünschen gemacht haben (*n* = 632) wurden gefragt, was sie im Umgang damit als hilfreich empfanden. Die meisten sahen ihre Menschenkenntnis (78 %; 490/632) sowie die Konsultation anderer Berufsgruppen (74 %; 466/632) als hilfreich an. Auch die Beratung im Team (58 %; 365/632) und Supervision (24 %; 151/632) wurden als Hilfe empfunden. Nur 4 % (23/632) gaben an, dass ihnen „nichts“ helfe. 9 % der Mitarbeitenden (58/632) ergänzten weitere berufliche Hilfen wie Kenntnisse zu Suizidwünschen (*n* = 16) oder ihre jeweilige Ausbildung (*n* = 13) und persönliche Ressourcen wie Spiritualität bzw. Weltanschauung (*n* = 6) oder familiären und sozialen Rückhalt (*n* = 5) im Umgang mit Suizidwünschen.

### Bedarf an institutioneller Unterstützung.

Die Mitarbeitenden wünschten sich als Unterstützung ihrer Institution mehrheitlich Fortbildungen (68 %; 505/741) sowie einen Kommunikationsleitfaden zu Suizidwünschen (55 %; 405/741). Auch der Bedarf an allgemeinen Informationen (39 %; 291/741), Supervision (36 %; 270/741) und Austauschforen (19 %; 138/741) wurde angegeben. Im Freitext wurde von 7 % (51/741) der Mitarbeitenden weiterer Bedarf an Unterstützungsangeboten genannt, der den Kategorien „für Mitarbeitende“ und „für Betroffene“ zugeordnet werden konnte. Am häufigsten wurde hierbei der Bedarf an konkreten *Ansprechpartner:innen für Mitarbeitende* (*n* = 14) und an einer *Beratungsstelle für Betroffene* (*n* = 5) angegeben.

### Bedarf an institutioneller Unterstützung spezifisch zum Urteil des Bundesverfassungsgerichts.

Die Mehrheit sah einen Informationsbedarf bezüglich des Urteils des Bundesverfassungsgerichts und wünschte sich Informationsveranstaltungen (62 %; 458/741), Informationsmaterial (61 %; 454/741) und Austauschforen zum Thema Suizidbeihilfe (33 %; 242/741). Weiterer Bedarf wurde von 6 % der Mitarbeitenden (43/741) in den Freitexten ergänzt. Zum Beispiel nannten sie den Bedarf an einer *Rechtsberatung* (*n* = 9), *Ansprechpartner:innen* (*n* = 8) oder konkreten *Handlungsempfehlungen* (*n* = 5) für Mitarbeitende.

### Kenntnisse zu Angeboten der Suizidprävention.

Kenntnisse zu Angeboten der Suizidprävention wurden von 33 % (246/741) der Mitarbeitenden bejaht; 56 % (417/741) kannten keine Angebote zur Suizidprävention. Keine Angabe machten 78 Mitarbeitende. Von den Mitarbeitenden, die Angebote der Suizidprävention kennen, wurden von 83 % (204/246) Beispiele genannt. Hierzu gehörten: *psychosoziale Beratungs- und Unterstützungsangebote* (Telefon- und Onlineberatung, Krisendienste und andere Hilfsangebote), *Angebote der Krankenversorgung und öffentliche Gesundheitsdienste* (u. a. Sozialpsychiatrischer Dienst, Psychiatrie, Seelsorge, Psychotherapie, Palliativmedizin) und *spezifische Maßnahmen* (u. a. Gespräche, Medikation) zur Suizidprävention.

### Ethisch begründete Position der Mitarbeitenden.

**Abb. 2:**
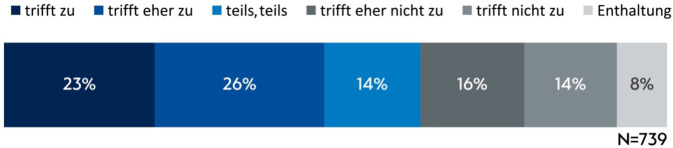
Wie bewerten Sie die folgende Aussage: „Durch die aktuelle gesellschaftliche Diskussion sehe ich mich persönlich herausgefordert, eine eigene ethisch begründete Position in der Diskussion zum assistierten Suizid zu finden.“

Durch die gesellschaftliche Diskussion zum Thema assistierter Suizid sieht sich knapp die Hälfte der Mitarbeitenden (49 %; 357/739) herausgefordert, eine eigene, ethisch begründete Position zu finden (Antworten „trifft zu“ und „trifft eher zu“, Abb. 2). Es enthielten sich 58 Mitarbeitende oder sie ließen diese Frage aus.

### Offene Mitteilungen zum Thema.

Die Möglichkeit der offenen Mitteilung wurde von 91 Mitarbeitenden genutzt. Wir identifizierten 4 übergeordnete Kategorien: *Berichte, Meinungen, Wünsche* und *Hinweise.*

In kurzen *Berichten* beschrieben Mitarbeitende ihre Berührungspunkte mit Todeswünschen, Suizidwünschen oder der Beihilfe zum Suizid. Einige Mitarbeitende teilten ihre *Meinung* zum assistierten Suizid mit. Hierbei wurde vor allem dem Recht auf Selbstbestimmung eine hohe Relevanz zugesprochen. Zudem kamen Ambivalenz und Ablehnung zur Sprache. Ergänzend zu den *Wünschen* bezüglich einer institutionellen Unterstützung der Mitarbeitenden, wurde Bedarf an einer gesetzlichen Regelung geäußert. Repräsentative Zitate sind in Tab. 1 aufgeführt.

**Tab. 1:** Repräsentative offene Mitteilungen in den Kategorien Berichte, Meinungen und Wünsche

Kategorie	Repräsentatives Zitat
**Berichte**	„Ich wurde schon häufig von Patient:innen gefragt, ob ich nicht etwas machen könne, damit es schneller zu Ende geht …“ (ASuPat-M668)
	„Einer [unserer] Patienten hat sich … Suizidassistenz besorgt und Freitod umgesetzt. Diese Erfahrung stellt meine ursprüngliche Ablehnung des ass. Suizids … in Frage.“ (ASuPat-M666)
	„In 14 Jahren … habe ich mehrfach miterlebt, wie der Sterbewunsch des Patienten nicht ernst genommen wurde.“ (ASuPat-M622)
**Meinungen**	„Selbstbestimmtes Sterben sollte genauso wichtig sein wie selbstbestimmtes Leben.“ (ASuPAt-M252)
	„… Was ist, wenn jemand die Entscheidung trifft, weil er dahingehend beeinflusst wurde? Wenn nicht körperliches Leiden, sondern materielle Bedenken zur Entscheidung führen …“ (ASuPat-M325)
	„Angesichts der zur Verfügung stehenden Mittel … empfinde ich eine Suizidassistenz als nicht mit den ärztlichen Grundsätzen vereinbar.“ (ASuPat-M354)
	„Jeder darf sein Leben beenden, wenn selbst gewünscht! Solange niemand unbeteiligter davon beeinträchtigt wird (springen vom Haus oder vor die Bahn).“ (ASuPat-M224)
**Wünsche**	„Ich würde mir wünschen, dass es im Falle eines Suizidwunsches konkrete Vorschläge für ein Vorgehen gibt, das den Patient:innen gerecht wird (Begutachtung, Zweitmeinung, Durchführungshilfen, wenn entsprechend beurteilt)“ (ASuPat-M289)
	„[Eine] gesetzliche Regelung ist dringend erforderlich.“ (ASuPat-M164)
	„Angebote und Informationen spezifisch für ältere Patient:innen wären sehr hilfreich“ (ASuPat-M312)

## Diskussion

### Häufigkeit Suizidwünsche und Anfragen für Suizidassistenz

Die Mehrheit der Teilnehmenden hat Erfahrungen mit Suizidwünschen im Behandlungskontext gemacht und fast die Hälfte wurde bereits von Patient:innen um konkrete Suizidassistenz gebeten. Diese deutliche Präsenz von Anfragen zur Beihilfe zum Suizid wird auch von Mitarbeitenden einzelner Disziplinen wie der Palliativmedizin, Hämato‑/Onkologie, Psychiatrie, Psychotherapie und Psychosomatik wahrgenommen, wie Befragungen von 2015 sowie jüngst von 2021 bis 2023 der entsprechenden Fachgesellschaften zeigen konnten [5–7]. Die Konkretheit der Gesuche variiert hierbei. Mit Fragen zu Informationen bezüglich des assistierten Suizids sahen sich in einer Studie [5] mehr als die Hälfte (57 %) der Hämato‑/Onkolog:innen konfrontiert, etwas weniger (43 %) wurden in einer weiteren Studie von ihren Patient:innen nach ihrer grundsätzlichen Bereitschaft zur Unterstützung eines Suizids befragt [5, 8]. Über explizite Bitten um Beihilfe zum Suizid berichteten 35 % Psychiater:innen [6] und sogar bis zu 70 % der Mitarbeitenden der Palliativmedizin [7, 9]. Anfragen zum assistierten Suizid betreffen nicht nur erfahrene Mitarbeitende. Eine Umfrage unter jüngeren Ärzt:innen mit maximal 5 Jahren Berufserfahrung ergab, dass bereits ein Drittel um Beihilfe zum Suizid gebeten wurden [10].

### Ressourcenstärkung

Die überwiegende Mehrheit der Mitarbeitenden kommt mit Suizidwünschen im Behandlungskontext in Berührung. Das macht sie – so wie alle anderen Mitarbeitenden der Gesundheitsversorgung – zu wichtigen Akteur:innen der Suizidprävention [11]. Diese gilt es in ihren Ressourcen zu stärken. Als hilfreich im Umgang mit Suizidwünschen wurden neben Menschenkenntnis auch die Möglichkeit zur Konsultation anderer Berufsgruppen, die Beratung im Team und Supervision empfunden, Ressourcen, die auch beim professionellen Umgang mit Tod und Sterben relevant sind [12, 13]. Interdisziplinäre und multiprofessionelle Teamstrukturen sowie eine regelmäßige Supervision gilt es explizit zu fördern, wie es die nationale Suizidpräventionsstrategie vorsieht [14].

### Diskrepanz zwischen Kenntnis der Strukturen der Suizidprävention und dem Bedarf

Trotz des hohen Bedarfs konnten Hilfsangebote zur Suizidprävention von vielen Mitarbeitenden nicht benannt werden. Dies deckt sich mit der Selbsteinschätzung des Wissensstands zum Thema Suizidalität und Suizidprävention auf Institutionsebene: Etwas mehr als ein Drittel (36 %) der befragten Kliniken, Verbände oder Institutionen der Krankenversorgung schätzten das eigene Wissen als schlecht ein oder enthielten sich [11]. Dies zeigt, dass die Etablierung und Stärkung von Strategien der Suizidprävention sowie deren flächendeckende Bekanntmachung weiterhin eine hohe Priorität bleiben.

### Bedarf an institutioneller Unterstützung: Verankerung suizidpräventiver Maßnahmen

Als Bedarf an institutioneller Unterstützung im Umgang mit Suizidwünschen wurden von den Mitarbeitenden Fortbildungen, Kommunikationsleitfäden und konkrete Ansprechpartner:innen sowie Verfahrensanweisungen angegeben. Zur Förderung der Suizidprävention in der medizinischen Versorgung bildet die Fort- und Weiterbildung eine der wesentlichen konkreten Maßnahmen [11, 14]. Hier besteht jedoch insbesondere in der somatischen Medizin ein nicht ausreichend gedeckter Bedarf, wie u. a. auch Untersuchungen zu Schulungen zum Umgang mit Todeswünschen zeigen konnten, in denen die Nachfrage das Angebot um das Doppelte übertraf [11, 15]. Fort- und Weiterbildungsmaßnahmen können Pflegepersonal, Ärzteschaft sowie anderen Berufsgruppen helfen, die eigene Haltung zum Thema zu reflektieren, und nachhaltig Wissen, kommunikative Fertigkeiten und Selbstsicherheit im Umgang mit Todeswünschen vermitteln [15]. Auch wenn Todeswünsche nicht mit Suizidwünschen gleichzusetzen sind, können sie doch dazu übergehen. Bereits in Studium und Ausbildung kann durch ergänzende Kursangebote der Umgang mit Suizidwünschen trainiert werden [16].

Es besteht weiterer Bedarf an formalisierten Arbeitshilfen in Form von Kommunikationsleitfäden und Verfahrensanweisungen sowie an der Benennung konkreter Ansprechpartner:innen, die im Umgang mit geäußerten Suizidwünschen beraten können. Übergeordnete Verfahrensanweisungen sind bereits in einigen Institutionen etabliert, wie beispielsweise am Universitätsklinikum Bonn [17]. Unterstützen können bereits etablierte Leitlinien, die das Thema Suizidalität oder den Umgang mit Todeswünschen aufgreifen [11, 18, 19]. Was den „Umgang mit Anfragen nach Assistenz bei der Selbsttötung“ betrifft, ist hierzu eine entsprechende S2k-Leitlinie in Arbeit [20].

### Informationen zum Urteil des Bundesverfassungsgerichts und zur rechtlichen Situation

Spezifisch zum Urteil des Bundesverfassungsgerichts und der gesellschaftlichen Debatte um die Beihilfe zum Suizid wünschten sich die Mitarbeitenden von ihrer arbeitgebenden Institution eine Bereitstellung von Informationen und einen Austausch zum Thema. Gerade eine Aufklärung bezüglich standesrechtlicher und gesetzlicher Grundlagen scheint hier besonders notwendig [7, 10].

### Selbstreflexion der eigenen Grundhaltung als Voraussetzung für eine offene Diskussion

Durch die gesellschaftliche Diskussion zum assistierten Suizid sieht die Hälfte der Mitarbeitenden eine Herausforderung darin, sich eine eigene, ethisch begründete Meinung zu bilden. Die konkreten Positionen wurden in unserer Umfrage nicht systematisch erfasst. Die qualitative Auswertung der offenen Mitteilungen ließ jedoch erkennen, dass sich diese – wie auch die gesellschaftliche Debatte – im Spannungsfeld zwischen Autonomie und Fürsorge bewegten. Im stationären Setting können klinische Ethikkomitees Diskursräume für Wertepluralität und unterschiedliche moralische Positionen schaffen und eine ethische Reflexion in Organisationen verankern.

### Limitationen

Als monozentrische Studie bildet unsere Umfrage einen Trend des Bedarfs der Mitarbeitenden einer einzelnen Institution der universitären Maximalversorgung in einer Großstadt ab. Zur Wahrung der Anonymität der Teilnehmenden wurde ihr Tätigkeitsbereich nicht erfasst, weshalb nicht ausgeschlossen werden kann, dass Mitarbeitende der Psychiatrie, Hämato-Onkologie, Palliativmedizin und Psychosomatik überproportional häufig vertreten sind. Zudem kann ein Selbstselektionsbias nicht ausgeschlossen werden, sodass die Untersuchung nicht repräsentativ für alle Mitarbeitenden ist. Eine weiterführende multizentrische Befragung unter Anwendung von quantitativen, aber gerade auch qualitativen Methoden (z. B. Interview) ggf. über einen längeren Zeitraum, eine separate Betrachtung einzelner Berufsgruppen sowie die Erfassung der genauen Tätigkeitsorte sind folgend sinnvoll.

## Fazit

Zusammenfassend sieht sich ein relevanter Teil der befragten Mitarbeitenden der stationären und ambulanten Krankenversorgung einer großen Universitätsklinik mit Suizidwünschen im Behandlungskontext konfrontiert. Nur rund ein Drittel kennt hingegen konkrete Angebote der Suizidprävention. Dies unterstreicht die Notwendigkeit, konkrete Strategien der Suizidprävention und Kenntnis darüber innerhalb der Einrichtung zu vermitteln und entsprechende Informationen zur Verfügung zu stellen. Es ist davon auszugehen, dass Mitarbeitende auch in vielen anderen Krankenhäusern, insbesondere in universitären Einrichtungen sowie in größeren und maximalversorgenden Häusern, in ähnlichem Maße mit Suizidwünschen konfrontiert werden und ebenfalls Unterstützung im Umgang damit benötigen. Eine gezielte Unterstützung der Mitarbeitenden ist erforderlich, um den Umgang mit Anfragen nach Suizidassistenz zu schulen, ihre Handlungskompetenz zu stärken und letztlich den Patient:innen gerecht zu werden.

## Supplementary Information

Fragebogen: Artikulation von Suizidwünschen im Behandlungskontext

## Data Availability

Die während der vorliegenden Studie erzeugten und analysierten Datensätze sind auf begründete Anfrage bei der Korrespondenzperson erhältlich.

## References

[1] Bundesverfassungsgericht (2020) Urteil des Zweiten Senats vom 26. Februar 2020 - 2 BvR 2347/15 -. https://www.bverfg.de/e/rs20200226_2bvr234715.htm. Accessed 13.3.2025

[2] Kremeike K, Pralong A, Bostrom K et al. (2021) ‘Desire to Die’ in palliative care patients-legal framework and recommendations of the national evidence-based guideline on palliative care in Germany. Ann Palliat Med 10:3594–3610. 10.21037/apm-20-381 33440974 10.21037/apm-20-381

[3] Eysenbach G (2004) Improving the quality of Web surveys: the Checklist for Reporting Results of Internet E‑Surveys (CHERRIES). J Med Internet Res 6:e34. 10.2196/jmir.6.3.e34 15471760 10.2196/jmir.6.3.e34 PMC1550605

[4] Mayring P (2014) Qualitative content analysis: theoretical foundation, basic procedures and software solution. https://www.ssoar.info/ssoar/handle/document/39517. Accessed 12.12.2024

[5] Schildmann J, Cinci M, Kupsch L et al. (2023) Evaluating requests for physician-assisted suicide. A survey among German oncologists. Cancer Med 12:1813–1820. 10.1002/cam4.4981 35770954 10.1002/cam4.4981 PMC9883542

[6] Wassiliwizky M, Gerlinger G, Domschke K, Reif A, Bader F, Pollmacher T (2022) Assisted suicide: Attitudes and experiences of members of the DGPPN. Nervenarzt 93:1134–1142. 10.1007/s00115-022-01391-2 36149457 10.1007/s00115-022-01391-2

[7] Schwartz J, Batzler YN, Schallenburger M et al. (2025) Dealing with assisted suicide-knowledge, attitudes and experiences of members of the German Association for Palliative Medicine. Bundesgesundheitsblatt Gesundheitsforschung Gesundheitsschutz 68:141–149. 10.1007/s00103-024-03960-z 39375218 10.1007/s00103-024-03960-z PMC11774969

[8] Schildmann J, Wünsch K, Winkler E (2015) Ärztlich assistierte Selbsttötung: Umfrage zur ärztlichen Versorgung von Krebspatienten. Ethische Überlegungen und Stellungnahme. https://www.dgho.de/publikationen/schriftenreihen/aerztlich-assistierte-selbsttoetung/dgho_schriftenreihe_Bd7-2015_web.pdf. Accessed 19.3.2025

[9] Turiaux J, Burner-Fritsch IS, Fegg M, Marckmann G, Bausewein C (2025) Anfragen zu und Praxis von Suizidassistenz – Ergebnisse einer Befragung unter Mitgliedern der Deutschen Gesellschaft für Palliativmedizin (DGP). Bundesgesundheitsblatt Gesundheitsforschung Gesundheitsschutz. 10.1007/s00103-025-04087-5 40569373 10.1007/s00103-025-04087-5 PMC12956932

[10] Kuppers R, Meier S, Batzler YN et al. (2024) Perspectives of a sample of mostly younger doctors on physician-assisted suicide. Bundesgesundheitsblatt Gesundheitsforschung Gesundheitsschutz 67:233–241. 10.1007/s00103-024-03833-5 38253871 10.1007/s00103-024-03833-5 PMC10834612

[11] Schneider B, Lindner R, Giegling I et al. (2021) Suizidprävention Deutschland – Aktueller Stand und Perspektiven. Deutsche Akademie für Suizidprävention e. V. (DASP). https://www.naspro.de/dl/Suizidpraevention-Deutschland-2021.pdf. Zugegriffen: 18.03.2025.

[12] Ateş G, Jaspers B (2020) Belastungs- und Schutzfaktoren in Teams der Hospiz- und Palliativversorgung in Nordrhein-Westfalen - eine Pilotstudie. https://alpha-nrw.de/wp-content/uploads/2020/06/studie_belastungs-schutzfaktoren_alpha_2020.pdf. Accessed 17.3.2025

[13] Herwest S, Kuhlmann SL, Willert AC, Ploner CJ, Kowski AB (2022) Burdens and Resources of Staff of a Specialized Ward for Neuropalliative Care: A Cross-Sectional Survey. Brain Sci 12(12):1697. 10.3390/brainsci12121697 36552156 10.3390/brainsci12121697 PMC9776069

[14] Bundesministerium-für-Gesundheit. (2023) Nationale Suizidpräventionsstrategie. https://www.bundesgesundheitsministerium.de/fileadmin/Dateien/5_Publikationen/Praevention/abschlussbericht/240430_Nationale_Suizidpraeventionsstrategie.pdf. Zugegriffen: 12.03.2025.

[15] Boström K, Dojan T, Frerich G et al. (2022) Umgang mit Todeswünschen in der Palliativversorgung – Evaluation eines Schulungsprogramms. Zeitschrift für Palliativmedizin 23:198–206. 10.1055/a-1729-7360

[16] Schallenburger M, Schwartz J, Batzler YN et al. (2024) Handling the desire to die- evaluation of an elective course for medical students. BMC Med Educ 24:279. 10.1186/s12909-024-05269-6 38494509 10.1186/s12909-024-05269-6 PMC10946106

[17] Bonn KEU (2023) Umgang mit Patientenwünschen oder -forderungen nach Suizidhilfe am Universitätsklinikum Bonn. https://www.ukbonn.de/site/assets/files/43403/sop_suizidhilfe.pdf

[18] Leitlinienprogramm Onkologie (Deutsche Krebsgesellschaft DK, AWMF) (2020) Palliativmedizin für Patienten mit einer nicht-heilbaren Krebserkrankung, Langversion 2.2, 2020 AWMF-Registernummer: 128/001OL. https://www.leitlinienprogramm-onkologie.de/leitlinien/palliativmedizin/. Accessed 19.3.2025

[19] Blumenthal S, Farr L, Fuchs S, Kahle C, Karl I, Lunden L. (2024) DEGAM S1-Handlungsempfehlung: Umgang mit dem Wunsch nach Suizidassistenz in der hausärztlichen Praxis. Deutsche Gesellschaft für Allgemeinmedizin und Familienmedizin e. V. https://register.awmf.org/de/leitlinien/detail/053-063. Zugegriffen: 7.04.2025.

[20] Bausewein C, Pollmächer T, Simon A, Wörmann B, Fuch S (2025) S2k-Leitlinie Umgang mit Anfragen nach Assistenz bei der Selbsttötung. AWMF online. https://register.awmf.org/de/leitlinien/detail/096-001. Accessed 13.3.2025

