# Supplementary material for: Articulation of patients’ suicidal ideation within the therapeutic context—experiences and needs of hospital healthcare professionals
Source: Bundesgesundheitsblatt Gesundheitsforschung Gesundheitsschutz. 2026 Feb 9;69(3):291–7. [Article in German] doi: 10.1007/s00103-026-04195-w (PMC12957639; doi:10.1007/s00103-026-04195-w)
Supplement: Supplementary file 1 — Fragebogen: Artikulation von Suizidwünschen im Behandlungskontext [file 103_2026_4195_MOESM1_ESM.pdf]

Fragebogen
Artikulation von Suizidwünschen im Behandlungskontext – Erfahrungen und Unterstützungsbedarf von Krankenhausmitarbeitenden

1. Wurde Ihnen gegenüber im Rahmen Ihrer beruflichen Tätigkeit schon einmal ein konkreter **Suizidwunsch geäußert**?
a. Ja, einmalig
b. Ja, circa 2- bis 5-mal
c. Ja, mehr als 5-mal
d. Nein
e. Weiß nicht/Keine Angabe

Frage 1 ist Filterfrage zu Frage 2

2. Was hilft Ihnen, im Rahmen Ihrer beruflichen Tätigkeit mit Suizidwünschen umzugehen? (Mehrfachnennung möglich)
a. Ihre durch Berufs- und Lebenserfahrung gewonnene Menschenkenntnis
b. Beratung im eigenen Team
c. Supervision
d. die Möglichkeit, andere Berufsgruppen hinzuziehen zu können (z.B. Psychiatrie, Psychologie, Seelsorge, Palliativteam, Fachkräfte der Sozialen Arbeit)
e. Nichts
f. Sonstiges (Freitext)

3. Welche Art der institutionellen Unterstützung wünschen Sie sich allgemein zum Thema Suizidwünsche? (Mehrfachnennung möglich)
a. Allgemeine Informationsveranstaltung zum Thema Suizidwünsche
b. Fortbildungen zur Kommunikation bei Suizidwünschen
c. Leitfaden zur Kommunikation bei Suizidwünschen
d. Austauschforen (Präsenz oder Online) zum Thema Suizidwünsche
e. Supervision
f. Sonstiges (Freitext)

4. Sind Sie im Rahmen Ihrer beruflichen Tätigkeit schon irgendwann von einem Patienten oder einer Patientin um **konkrete Unterstützung** bei der **Durchführung eines Suizids** gebeten worden?
a. Ja, einmalig
b. Ja, circa 2- bis 5-mal
c. Ja, mehr als 5-mal
d. Nein
e. Weiß nicht/Keine Angabe

5. Welche Art der institutionellen Unterstützung wünschen Sie sich spezifisch zum Urteil des Bundesverfassungsgerichts zum assistierten Suizid, den zugehörigen Gesetzentwürfen und der gesellschaftlichen Debatte? (Mehrfachnennung möglich)
a. Informationsveranstaltung
b. Austauschforen (Präsenz oder Online) zum Thema
c. Informationsmaterial
d. Ich habe keinen Informations- bzw. Unterstützungsbedarf
e. Sonstiges (Freitext)

6. Wie bewerten Sie die folgende Aussage? "Durch die aktuelle gesellschaftliche Diskussion sehe ich mich persönlich herausgefordert, eine eigene ethisch begründete Position in der Diskussion zum assistierten Suizid zu finden."
a. trifft zu
b. trifft eher zu
c. teils-teils
d. trifft eher nicht zu
e. trifft nicht zu
f. Weiß nicht/Keine Angabe

7. Kennen Sie Angebote zur Suizidprävention?
a. Ja
b. Nein
c. Weiß nicht/Keine Angabe

Filterfrage 7 für Frage 8
8. Wenn Ja, welche Angebote kennen Sie? (Freitext)

9. Möchten Sie uns noch etwas zum Thema mitteilen? (Freitext)

10. Welcher Berufsgruppe gehören Sie an?
a. Pflege
b. Ärzteschaft
c. Fachkräfte der Sozialen Arbeit
d. Psycholog:innen
e. Physiotherapeut:innen
f. Seelsorger:innen
g. Andere Berufsgruppe (Freitext)
